# Improving eel pass efficiency: The role of crest shape and water flow in facilitating upstream juvenile eel migration

**DOI:** 10.1111/jfb.70017

**Published:** 2025-04-11

**Authors:** Michael J. Williamson, Bryony E. Allen, Jack A. Brand, Charlotte Pike, Chris Sergeant, Chris Grzesiok, Rosalind M. Wright, Adam T. Piper

**Affiliations:** ^1^ Institute of Zoology Zoological Society of London London UK; ^2^ Department of Genetics, Evolution and Environment University College London London UK; ^3^ Department of Wildlife, Fish, and Environmental Studies Swedish University of Agricultural Sciences Umeå Sweden; ^4^ Conservation and Policy Zoological Society of London London UK; ^5^ Environment Agency, Sentinel House Fradley UK; ^6^ Environment Agency, Rivers House Feering UK

**Keywords:** *Anguilla*, crest, efficiency, European eel, fish migration, flow, hydrodynamics, pass

## Abstract

River connectivity is crucial for the European eel (*Anguilla anguilla*) to complete its complex life cycle, which is vital for upstream recruitment to the declining population of this critically endangered catadromous fish. Eel passes, or ladders, are frequently installed on riverine structures, such as dams and weirs, to mitigate barrier effects and restore connectivity for upstream migrating eel. Efforts to optimise the effectiveness of passes have previously focused on the ascent section, quantifying the effects of climbing substrate, longitudinal slope, lateral slope and flow rate. However, conditions at the pass crest also impact the rapidity and success of upstream movements. Using controlled experiments and custom‐built eel passes with contrasting crest shapes (curved vs. sloped) and flow directions (ascending vs. descending), we quantified the effect of crest conditions on the attempt success, passage efficiency and speed of ascending juvenile eel. Only three of the four treatments (sloped ascending, curved descending and curved ascending) demonstrated passage efficiencies significantly greater than 50%. Transit speed at the crest was significantly quicker (~3.5 min) in passes with a curved crest shape and ascending flow compared to the control. Our findings indicate that simple modifications to the shape of the pass crest and the configuration of flow delivery can help minimise delay and enhance passage efficiency. This, in turn, will increase upstream migration success and contribute to conservation and management goals, such as the EU Eel Regulation and The Eels (England and Wales) Regulations 2009.

## 1 INTRODUCTION

The European eel (*Anguilla anguilla*), a catadromous fish species of ecological and commercial importance, requires both continental and oceanic habitats to complete its complex life cycle (Arai, 2020; Drouineau et al., 2018; Grassi & Lankester, 1897; Righton et al., 2021; Schmidt, 1912; Tesch, 2003). This renders it susceptible to a variety of natural and anthropogenic threats (Drouineau et al., 2018; Jacoby et al., 2015). Consequently, there have been severe declines across its range over recent decades, with recruitment in 2023 estimated to be between 0.5% and 7.4% of 1960–1979 baseline levels (ICES, 2024). *A. anguilla* is now listed as critically endangered on the International Union for Conservation of Nature (IUCN) Red List of Threatened Species (Pike et al., 2020) and under Appendix II of the Convention on International Trade in Endangered Species of Wild Fauna and Flora (CITES) (CITES, 2007). In 2007, the European Union implemented the ‘Eel Regulation’ (EU Council Regulation number: 1100/2007), which requires European Union member states to develop and implement eel management plans (EMPs) to improve the status of the European eel. In England and Wales, measures to achieve these commitments are implemented through ‘The Eels (England and Wales) Regulations 2009’ (U.K. Government, 2009).

Continued recruitment to the declining panmictic population of *A. anguilla* is dependent on a series of migrations. Although fresh water is not required to complete their life cycle (Arai, 2022; Durif et al., 2023), the inter‐habitat, upstream migration from estuaries to freshwater habitats is an important migration route for juvenile eel (Arai, 2020; Arai, 2022; Enbody et al., 2021; Tesch, 2003). Anthropogenic riverine barriers, such as dams, weirs, hydropower facilities, pumping stations and tidal gates, can significantly restrict or completely prevent access to these important freshwater habitats (Drouineau et al., 2018; Jacoby et al., 2015; Laffaille et al., 2007; Winter et al., 2006). Delays to, or prevention of, upstream migration reduce recruitment into freshwater growing habitats and may directly impact eel survival (Halvorsen et al., 2020; Jellyman & Arai, 2016; White & Knights, 1997) through heightened predation (Matthews et al., 2001; McLaughlin et al., 2013; Trancart et al., 2018) and increased risk of parasitism and disease, as individuals accumulate at high densities below barriers (Garcia Leaniz, 2008; McLaughlin et al., 2013). A comprehensive assessment of stream fragmentation in Great Britain identified at least one artificial barrier every 1.5 km of stream (Jones et al., 2019). In England and Wales alone, there are an estimated 36,935 artificial obstructions, all of which have the potential to hinder upstream eel migration (https://river-obstacles-theriverstrust.hub.arcgis.com/).

Installing upstream eel passes, or ladders, is a commonly employed conservation tool to improve river connectivity for eel (Knights & White, 1998; Pecorelli et al., 2019; Piper et al., 2012; Watz et al., 2019) as is required by the Eels (England and Wales) Regulations 2009 (U.K. Government, 2009). Upstream eel passes attempt to mitigate barrier impacts by providing a migration route with comparatively favourable conditions for ascent at structures (Knights & White, 1998; Piper et al., 2012; Piper et al., 2023; Santos et al., 2016; Tremblay et al., 2016; Watz et al., 2019). Just as riverine barriers are highly variable, so too are the eel passes designed to alleviate their impacts. These passes range from the simple addition of artificial climbing substrate to the barrier face to technical up‐and‐over passes, which provide a migration route that completely circumvents the structure (Fjeldstad et al., 2018; Solomon & Beach, 2007). This latter type generally has a gradually inclining trough (ascent ramp) extending from downstream of the structure to or beyond its highest point. The trough is lined with a climbing substrate such as bristles or studs and culminates at a crest beyond which the migrating eel simply drop into the watercourse upstream of the structure or are conveyed there via a descent ramp (Solomon & Beach, 2007). Water is pumped to the pass crest where it typically splits, with a proportion directed down the ascent ramp as conveyance or attraction flow, and the remainder directed upstream of the crest apex to non‐volitionally convey eel down the descent section (Knights & White, 1998). Juvenile eel predominately use rheotactic and olfactory cues to orient themselves in lotic waterbodies (Briand et al., 2002; Du Bureau Colombier et al., 2007; Tosi et al., 1990). Their positive rheotactic behaviour is exploited within eel pass design both to stimulate climbing of the ascent section using conveyance flow (Haro, 2013; Jellyman et al., 2017) and to attract eel to the pass entrance using an additional attractant flow (Piper et al., 2012; Watz et al., 2019).

There is wide variation in the reported efficiencies of upstream eel passage facilities, with robust tests of installed passes still lacking and concern that some may function poorly (Drouineau et al., 2018; Padgett et al., 2020). Previous quantitative assessments have primarily focused on the ascent ramp, exploring the effects of climbing substrate type, conveyance flow rate and longitudinal and lateral slope angles on transit times and passage success (Ibnu Syihab et al., 2021; Padgett et al., 2020; Piper et al., 2023; Vowles et al., 2015; Vowles et al., 2017; Watz et al., 2019). Little to no attention has been directed towards the next stage of transit when an ascending eel reaches the end of the ascent ramp and encounters the pass crest. Field observations by the authors (A.T.P., C.G. and R.M.W.) and others (Rosewarne & Wright, 2024) suggest that the complex flow conditions created where flow splits at the crest may serve to disorientate eel, thereby delaying or even preventing passage. For example, eel have been observed reaching the crest, pausing, exploring, volitionally turning and commencing a descent of the ascent ramp. An effective fish pass should facilitate passage without inducing delay, stress, disease or injury, and without demanding additional energy expenditure (Castro‐Santos et al., 2009; Fjeldstad et al., 2018). A focus within the continuing optimisation of eel passes should therefore be to create conditions that encourage migrants to advance through all sections of the pass in a linear manner, without delays and passage rejection.

In the current study, we tested the effect of modifying both the shape of the crest and flow direction at the crest on eel passage efficiency using custom‐built eel passes within controlled laboratory trials. Findings from this, the first study to our knowledge that specifically addresses the crest section of upstream eel passes, will contribute knowledge towards optimising the design of technical eel passes for juvenile eel and help inform ongoing efforts to improve the functioning of installed and future passes and increase recruitment of juvenile European eel to upstream habitats.

## 2 MATERIALS AND METHODS

### 2.1 Ethics statement

The care and use of experimental animals complied with UK animal welfare laws, guidelines and policies. All procedures were subject to ethical approval by the Zoological Society of London Ethics Committee and conducted under Home Office licence (Establishment Licence XBABDAACB).

### 2.2 Eel capture and husbandry

Juvenile eel for trials were sourced by the Environment Agency from up‐and‐over pumped passes with monitoring traps at two sites in the UK from July to October 2022, (a) Brownshill Sluice, Great Ouse, Cambridgeshire (52.3358° N, 0.0086° E) and (b) Judas Gap, River Stour, Suffolk (51.9549° N, 1.0257° E). Captured eel were transported (maximum 2 h) to the Institute of Zoology, Zoological Society of London, in tanks of aerated river water and subsequently transferred together to an aerated holding tank (minimum water volume 250 L, maximum 10 g 50%/L), with gradual water acclimation achieved by incrementally replacing 50% of the transport tank water with holding facility water over a 1‐h period. A subsample of 30 eel from each batch was transported to the Environment Agency's fish health laboratory to undergo a sacrificial health check, which included parasitology [e.g., whitespot (*Ichthyophthirius multifiliis*), *Myxidium giardia*, *Anguillicoloides crassus*)], virology [e.g., Anguillid herpesvirus (AngHV‐1), Eel Virus European (EVE)] and histology. If pathogen levels in the subsample were deemed by fish health experts to be normal or lower than normal for a natural population, the corresponding batch was used in trials.

Experimental subjects were acclimated to laboratory conditions for a minimum of 24 h before the commencement of trials and fed a maintenance diet of one cube of each defrosted bloodworm, daphnia and brine shrimp (Aquadip, UK) and 1.3 g of algae wafers (Hikari, UK) per 500 eel every other day. Water temperature was maintained at 17 ± 1°C, and other key water quality parameters (dissolved oxygen, ammonia and pH) were monitored every 30 min (Seneye reef, Seneye) to ensure that they remained within acceptable limits (minimum 80% oxygen saturation; maximum 1.0 mgL^−1^ ammonia; pH 6–8). Holding tanks were fitted with solid lids to prevent eel escape and possible disturbance caused by personnel entering and leaving the room. However, low‐level ambient light from laboratory lighting was able to enter the tank around the lid perimeter and pipe entry ports. Photoperiod was maintained at 12 h (8:00 AM – 8:00 PM) during British Summer Time (27 March 2022–30 October 2022) and altered to a 10‐h photoperiod when daylight savings finished on 30 October 2022. A water change (40%–50%) was conducted every other day, alternated with feeding days. Water was obtained through reverse osmosis (80%–90%) remineralised with tap water (10%–20%) and dechlorinated (Liquid Filter Medium, Aquadip). All checks and husbandry tasks, which involved opening the holding tank lids, were conducted in darkness with the use of red‐light torches. All husbandry equipment was washed and thoroughly dried between uses to kill any pathogens. Hold time of eel on‐site was limited to 7 days.

### 2.3 Experimental set‐up

**FIGURE 1:**
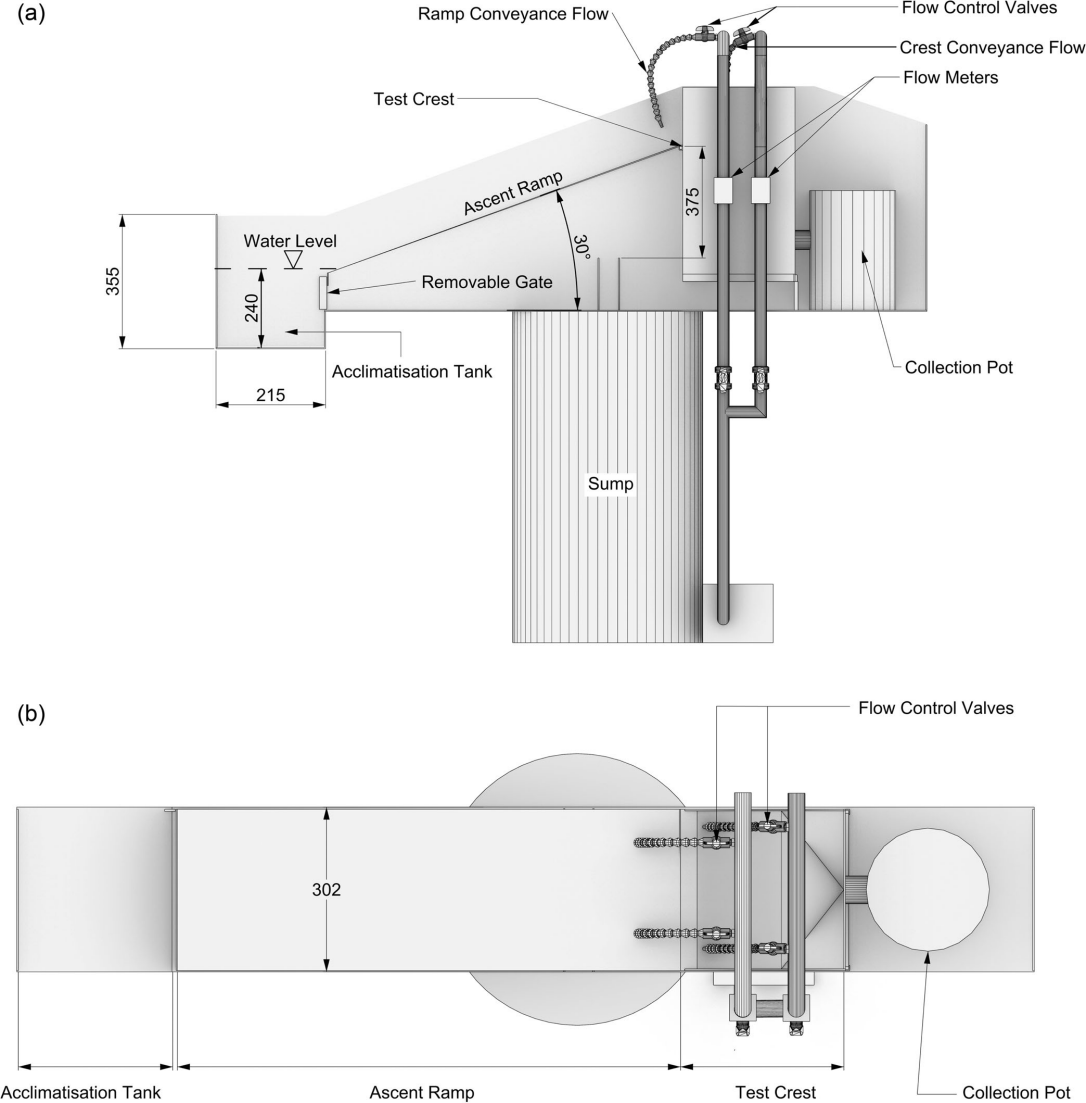
The generalised pass set‐up. Dimensions in millimetre, viewed from (a) the side and (b) overhead.

**FIGURE 2:**
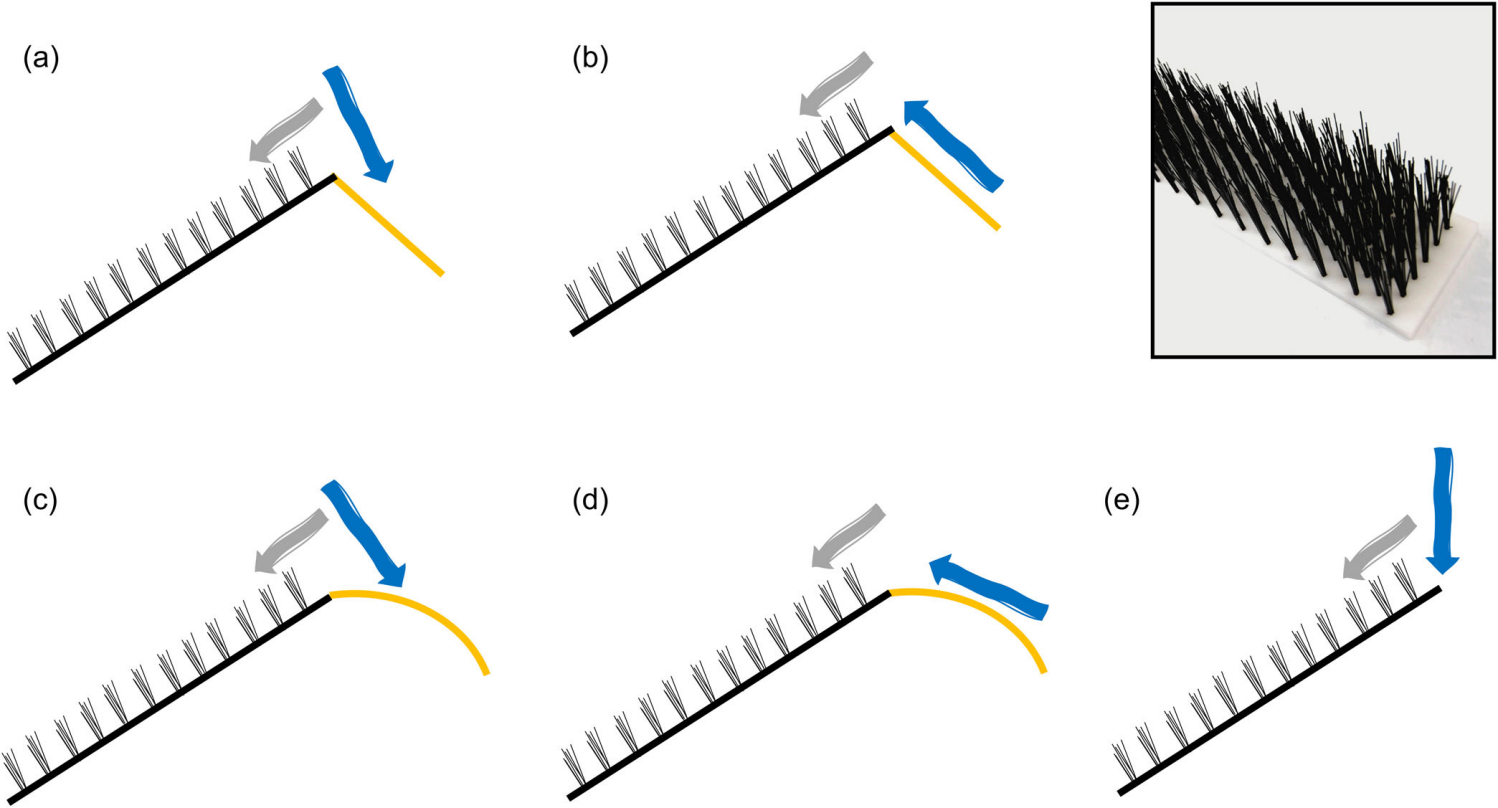
Schematic of the five crest configurations. (a) Sloped + descending crest flow, (b) sloped + ascending crest flow, (c) curved + descending crest flow, (d) curved + ascending crest flow and (e) control, where the descent section is depicted by yellow lines, crest flow by blue arrows and ascent ramp conveyance flow by grey arrows. Inset photograph shows the bristle climbing substrate.

Lengths of juvenile eel used in the study ranged from 61 to 158 mm (mean = 91 ± 11.2, ± standard deviation). Trials were conducted over 36 days during the period from 13 July to 8 November 2022 using five custom‐built eel passes (designed by the Environment Agency and manufactured by Hydrotec Consultants Ltd., UK) (Figure 1). All passes had an ascent ramp (W 302 mm, L 1000 mm, 30° longitudinal slope) fitted with a climbing substrate of nylon bristle clusters with 20‐mm spacing (Cottam Brush Ltd., UK) (Figure 2, inset). Two of the passes had a sloped descent section (200 mm length, 40° slope) beyond the crest apex (Figure 2a,b), two had an arc‐shaped curved descent section (200 mm length, radius 30 mm) (Figure 2c,d) and one had no descent section (control) to simulate a facility with no structural support beyond the crest (Figure 2e). For more detail on the experimental set‐up, see Supporting Information Appendix S1.

### 2.4 Experimental procedure

Trials were conducted during daytime (09:00 AM–05:00 PM). Juvenile eel migration occurs during both day and night, but activity is greatest during the first few hours of darkness (Bardonnet et al., 2003; Harrison et al., 2014 and references therein). During trials, the facility was lit exclusively with infra‐red light to illuminate for filming, and, during trial set‐up (ca. 30 min), the facility was lit with red light; both are outside the spectral sensitivity of European eel (650–850 nm wavelengths) (Archer et al., 1995; Hope et al., 1998). Prior to trials, test individuals were removed from the holding tank by random sweeps of a hand‐net at all heights in the water column and transferred to a tray. To reduce handling time, eel were visually size‐matched into groups of 20 and then transferred to storage tubes with mesh ends (21 cm length, 5 cm diameter) and returned to the holding tank until the trial (≤ 15 min). The flow circulation pumps were started, and each group of eel was transferred within the holding tube and released into the acclimatisation tank (Figure 1) to acclimate for 15 min. The trial started when the mesh gate at the bottom of the ascent ramp was lifted.

**FIGURE 3:**
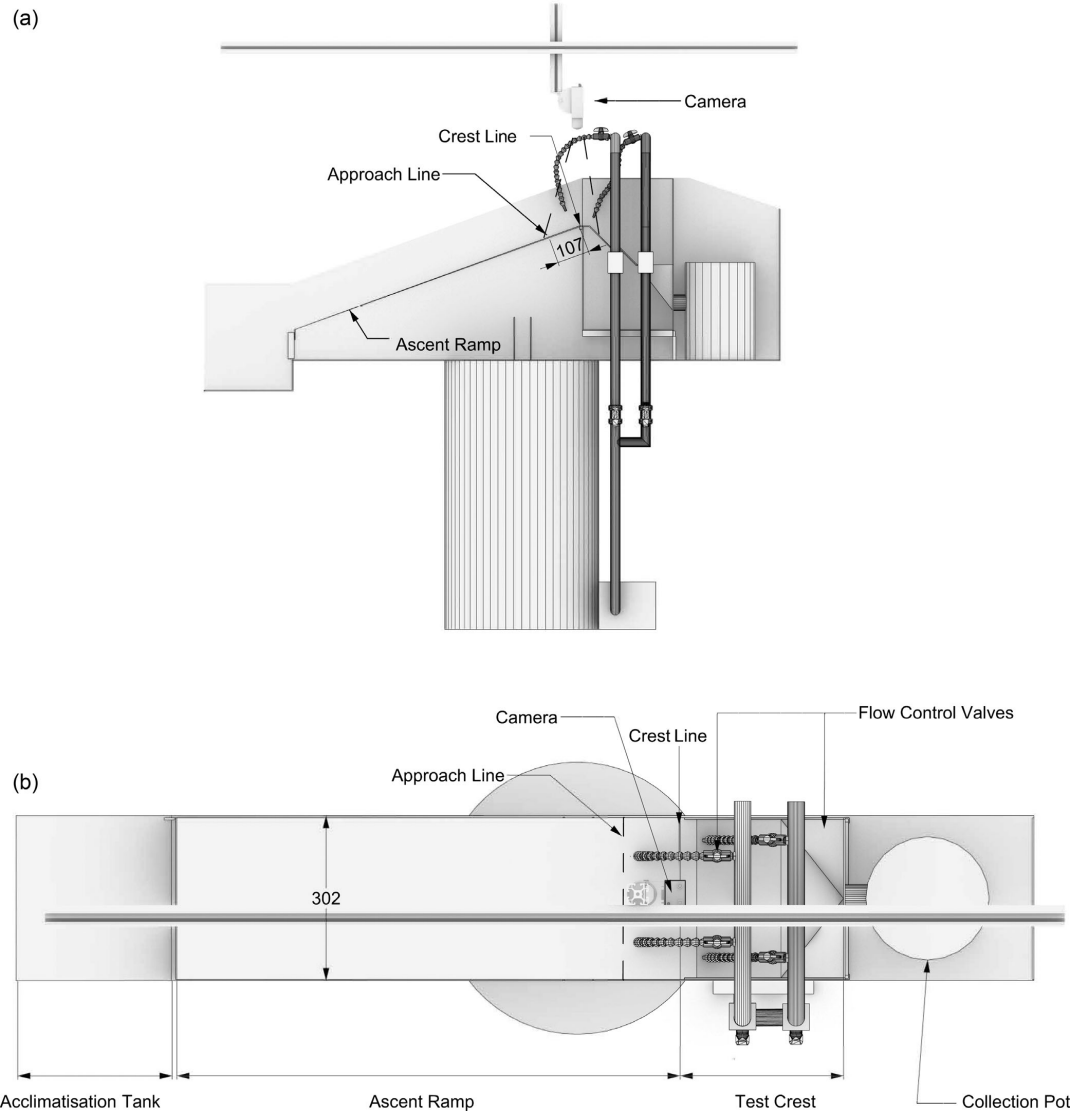
Diagram of the eel pass camera configuration. The figure shows the region of the pass covered by the infra‐red camera, viewed from (a) the side and (b) overhead.

Each trial lasted 2 h. Between three and five passes were operated simultaneously, and the ascending and descending crest flow directions were randomly assigned to the sloped and curved crest shapes. The control pass was operated during every batch of trials. Eel movements as they navigated the pass were continuously recorded at 25 fps using infra‐red‐sensitive cameras (HDCC500, Abus and SDN‐550, Samsung) and a video recorder (HD Analogue Recorder HDCC9001, Abus). A camera on each pass was positioned to capture behaviour on the crest and just beyond it (Figure 3).

On completion of the trial, the pumps were switched off, and any eel remaining on the ascent ramp were manually washed back into the acclimatisation tank. Individuals that successfully navigated the ascent ramp and crest exited the pass into a flow‐through tank connected to a removable collection pot (Figure 1). Eel from the collection pot (successful migrants) and the acclimatisation tank (unsuccessful migrants) were counted and measured. Individuals were used only once and held for a maximum of 7 days in the facility before being released into the wild close to the site of capture (Supporting Information Appendix S1).

### 2.5 Quantifying passage metrics and eel behaviour

Video footage files were assigned an anonymised code and then manually analysed using VLC media software (https://www.videolan.org/) by a naïve technician who had no prior knowledge of the study treatments. Five percent of footage was randomly spot‐checked for analysis errors and consistency confirmed among trials. The times when individuals reached relevant points in the pass were extracted: (a) the approach line, 107 mm downstream of the crest apex; (b) the crest line, that is, the intersection between the ascent ramp and crest; and (c) completed the crest structure, that is, reached the point of no return on the crest (Figure 3). Test subjects were not individually identifiable in the footage; therefore eel that moved back downstream of the approach line, either by volitional rejection or non‐volitional wash back, and then reascended were scored as a separate attempt. Eel attempts were classed as a success if the eel successfully navigated the crest structure, or a failure if they descended below the approach line or did not fully navigate the crest structure before the end of the trial. The following metrics were calculated for each passage attempt: (a) transit time from approach line to the crest (TTA‐C) and (b) transit time from reaching the crest to successful transition completion (TTC‐S) (Table 1).

**TABLE 1:** Description of the passage points and metrics used to determine European eel passage performance and behaviour in test passes under varying crest shape and crest flow direction, and the explanatory variables used in modelling.

Passage points’ definition	
Attempt	Ascended beyond the approach line, a point 107 mm downstream of the crest line, into the pre‐crest area of interest (Figure 3).
Crest	The first instance the eel reaches the crest line, the intersection between the ramp and crest, after crossing the approach line (Figure 3).
Crest success	The completion of the crest and thus successful passage of the whole pass.
Failure	An attempt that does not result in successful upstream passage over the crest, including volitional rejections and washdown descents below the approach line. Also, those individuals remaining on the pass, above the approach line, at the end of the 2‐h trial. Individuals may fail before or after reaching the crest line.

Metrics/response variables’ definition	
Attempt success	If an attempt was successful, it was coded 1; if unsuccessful, it was coded 0.
Passage efficiency	The proportion of total passage attempts by eel that were successful within a group.
Transit time from approach to crest (TTA‐C)	Time between arrival at the approach line (start of attempt) and first contact with the crest line.
Transit time from crest to success (TTC‐S)	Time between first contact with the crest line and successful completion of the pass, as a measure of time spent on the crest (crest delay).

Explanatory variables’ definition	
Treatment group	Combination of crest shape and crest flow type (four treatments and control)
Trial	A single 2‐h period where two to five groups of 20 eel were released onto passes of varying treatments
Trial number	Sequential number of trials
Order of trial	The order of the trial within day (first, second or third)
Hold time (days)	Number of days since arriving on‐site
Start ramp conveyance flow rate (L min^−1^)	The ramp conveyance flow rate at the start of a trial
Start crest conveyance flow rate (L min^−1^)	The crest conveyance flow rate at the start of a trial
Start water temperature (°C)	Temperature of water in the pass at the start of the trial
Pass ID (random effect)	Individual identifier of pass used (five in total)
Group ID (random effect)	Combination of trial number and pass ID to generate a unique identifier for each group of 20 eel tested

### 2.6 Statistical analysis

All analyses were conducted in R version 4.4.2 (R Core Team, 2024). Treatment groups with a temperature difference between the start and end of the trial greater than or equal to 1°C were removed from the analysis (3.8% of observations). Similarly, trials with crest flow rates >1.5 L min^−1^ below the 15 L min^−1^ target were removed prior to analysis (13.2% of observations) so that final crest flow rates ranged between 14.5 and 16.1 L min^−1^. In all cases, we fitted Bayesian generalised linear mixed models using the *brms* package (Bürkner, 2017). Post hoc comparisons were performed using a combination of the *emmeans* (Lenth, 2024), *modelbased* (Makowski et al., 2020) and *marginaleffects* (Arel‐Bundock et al., 2024) packages. For analysis, crest shape type and crest flow type were combined, yielding four treatments and the control (Figure 2). In all models, explanatory variables with continuous distributions were rescaled (Harrison et al., 2018) using the ‘scale’ function from the *base* package (R Core Team, 2024) to aid model fitting and interpretation. All models were run on four chains using weakly informative priors for a total of 3000 iterations (warm‐up = 1000 iterations). Posterior predictive checks were performed to ensure adequate model fits, and the examination of trace plots and the Gelman‐Rubin diagnostic statistic indicated that models had converged with minimal among‐chain variability (*R̂* = 1.00). We report posterior medians with 95% credible intervals (CI). Clear evidence for an effect was considered when CIs did not overlap with zero.

For analyses, models were chosen a priori based on the hypothesis of interest and key variables that we wanted to hold constant. To investigate treatment differences in the probability of a passage attempt being successful, we included attempt success as a binary response variable (0 or 1) with a Bernoulli distribution. Hold time and order of trial were included as continuous covariates, treatment group as a fixed‐effect factor (5 levels) and pass ID and group ID as random intercepts. As group ID includes trial date, neither date nor other temporal variables such as season were included in the model. Second, we investigated the proportion of total attempts within each group of ~20 eel that were successful as a measure of passage efficiency using a zero–one‐inflated beta distribution. We allowed phi to vary among treatment groups. Treatment group was included as a fixed‐effect factor, whereas hold time and order of trial were included as continuous covariates. As individual eel could not be identified from the video footage, length was not included as a fixed effect. Pass ID was included as a random intercept to account for repeated measures (group ID was not included as a random intercept because this analysis was conducted at the group level). To assess whether the majority of attempts within a trial were successful, treatment group contrasts were applied against a benchmark of 50% passage efficiency using the *hypothesis()* function from the *brms* package (Bürkner, 2017). Finally, to investigate the time taken to transit from the approach line to the crest and time taken to successfully pass the crest, we fitted two separate models for the response variables TTA‐C and TTC‐S with exponential distributions. The fixed‐effects structure was the same as that described above. Pass ID and group ID were also included as random effects.

## 3 RESULTS

### 3.1 Overall passage success

Counts of eel in the collection pots at the end of trials indicated that between 1 and 20 eel successfully navigated the whole pass structure. Median overall passage success ranged from 8 eel (range 1–19) in the curved ascending treatment to 14 eel (range 4–20) in the sloped ascending treatment.

### 3.2 Probability of attempt success

**FIGURE 4:**
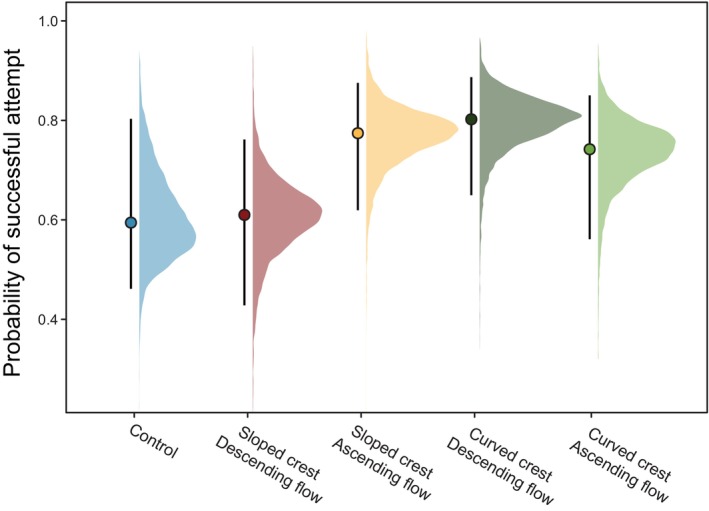
Predicted probability of successful attempt of *Anguilla anguilla* for each treatment and the control, generated by a Bayesian generalised model. Coloured distributions display the respective posterior distributions for each treatment group. Point estimates represent posterior medians, whereas error bars denote 95% credible intervals (CIs).

A total of 2408 ascent attempts were recorded (Table 2). Of these, 1602 (67%) were successful, whereby eel navigated both the ascent ramp, the crest and reached the collection pot. Predicted probabilities of a single passage attempt being successful ranged from 0.59 to 0.8, with the sloped ascending, curved descending and curved ascending treatment groups all demonstrating marginally, although non‐significantly, increased predicted probabilities of a single passage attempt being successful compared to the control (Figure 4; Table 3). There was also a negative effect of hold time, with eel that had been in the laboratory for longer having a decreased probability of successful attempts (estimate [95% CI] = −0.30 [−0.57, −0.04]). Credibility intervals can be found in Figure S1A.

**TABLE 2:** Summary statistics of passage metrics from video footage for trials broken down by crest shape and crest flow treatment.

Treatment group	Number of groups analysed	Total number attempts	Total number of successes	Total number of failures	Overall % attempt success
Control	51	690	356	334	51.6
Slope descending	35	468	289	179	61.8
Slope ascending	37	486	372	114	76.5
Curved descending	33	429	340	89	79.3
Curved ascending	34	335	245	90	73.1

**TABLE 3:** Predicted median [± 95% credible interval (CI)] probability of an attempt being successful for each treatment and the control, generated by a Bayesian generalised model.

Treatment combination	Predicted probability [95% CI]
Control	0.59 [0.46, 0.80]
Sloped descending	0.61 [0.43, 0.76]
Sloped ascending	0.77 [0.62, 0.88]
Curved descending	0.80 [0.65, 0.89]
Curved ascending	0.74 [0.56, 0.85]

Treatment contrasts	Contrast [95% CI]
Control – sloped descending	−0.05 [−0.72, 1.06]
Control – sloped ascending	−0.83 [−1.52, 0.30]
Control – curved descending	−0.99 [−1.70, 0.17]
Control – curved ascending	−0.65 [−1.35, 0.52]

*Note*: Contrasts are reported on the link scale.

### 3.3 Crest passage efficiency

**FIGURE 5:**
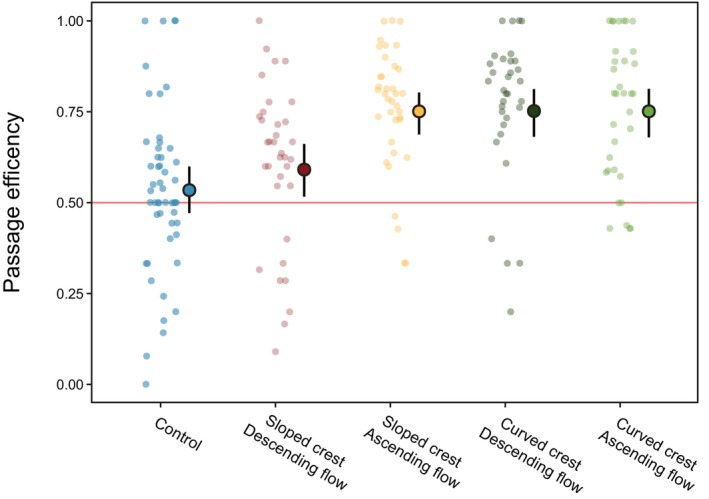
Actual and predicted passage efficiency of *Anguilla anguilla* (proportion of total attempts that are successful) within each trial for each treatment and the control. Coloured points represent the underlying data, whereas point estimates represent posterior medians [± 95% credit intervals (CI)] extracted from the Bayesian generalised linear model.

The sloped ascending, curved descending and curved ascending treatment groups all demonstrated a marginally greater proportion of total attempts that were successful within each group, that is, higher passage efficiency than the control and sloped descending, although CIs of the treatment contrasts slightly overlapped zero (Table 4). However, although the predicted passage efficiency was significantly higher than 50% in the sloped ascending [contrast (95% CI) = 0.22 (0.10, 0.32)], curved descending [contrast (95% CI) = 0.22 (0.08, 0.32)] and curved ascending [contrast (95% CI) = 0.18 (0.03, 0.29)] treatment groups, this was not the case for either the sloped descending treatment [contrast (95% CI) = 0.07 (−0.07, 0.19)] or the control [contrast (95% CI) = 0.05 (−0.07, 0.22)] (Figure 5; Table 4). CIs can be found in Figure S1B.

**TABLE 4:** Predicted proportion [± 95% credible interval (CI)] of total attempts that were successful within each group, generated by a Bayesian generalised model.

Treatment combination	Predicted proportion [95% CI]
Control	0.53 [0.47, 0.60]
Sloped descending	0.59 [0.52, 0.66]
Sloped ascending	0.75 [0.69, 0.80]
Curved descending	0.75 [0.68, 0.81]
Curved ascending	0.75 [0.68, 0.81]

Treatment contrasts	Contrast [95% CI]
Control – sloped descending	−0.02 [−0.16, 0.19]
Control – sloped ascending	−0.19 [−0.31, 0.02]
Control – curved descending	−0.19 [−0.32, 0.00]
Control – curved ascending	−0.19 [−0.31, 0.01]

Contrast: are proportions of success higher than 50%?	Contrast [95% CI]
Control	0.05 [−0.07, 0.22]
Sloped descending	0.07 [−0.07, 0.19]
Sloped ascending	0.22 [0.10, 0.32]
Curved descending	0.22 [0.08, 0.32]
Curved ascending	0.18 [0.03, 0.29]

*Note*: Treatment contrasts are reported on the link scale.

### 3.4 Transit times

**FIGURE 6:**
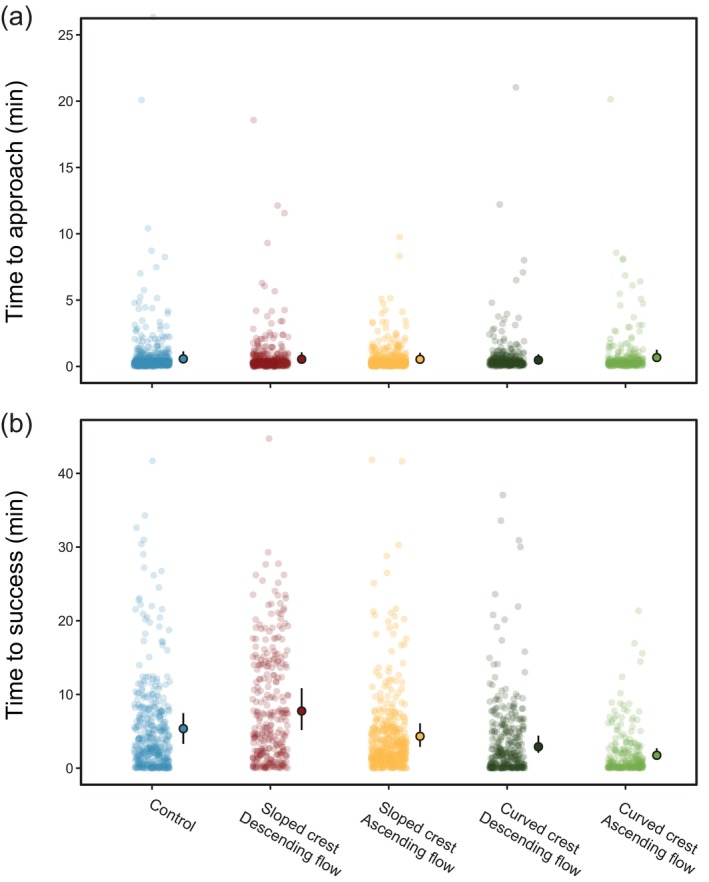
Actual and predicted median transmit time (in minutes) of *Anguilla anguilla* on eel pass trials. From (a) the approach to the crest and (b) the first point of contact with the crest to successfully completing the pass for each treatment and the control. Point estimates represent posterior medians, whereas error bars denote 95% credible intervals (CIs). Raw data are displayed with small, open circles. The cartesian coordinates of the plots were restricted to exclude five outliers in plot (a) and 12 outliers in plot (b). This was done to better visualise treatment‐level differences in median transit time. However, these outliers were included in all models and are accounted for in model estimated posterior medians and CIs that are displayed on the plots.

There were no pair‐wise differences among treatment groups and the control in transit time from the approach line to the crest, and there was little evidence for the effects of any other explanatory variables (all CIs overlapped zero) (Figure 6a; Table S1). However, there were differences in the predicted transit time from the crest to the point of successful passage, with eel in the curved ascending treatment group on average ~3.5 min quicker to successful passage than those in the control (Figure 6b; Table 5). Eel from the curved descending group were also marginally faster (~2.5 min) when compared to the control, although CIs very slightly overlapped zero (Figure 6b; Table 5). There was little evidence for substantial differences in transit time to success between the control and either the sloped descending or the sloped ascending treatments (Figure 6b; Table S5). Credibility intervals can be found in Figure S1C,D.

**TABLE 5:** Predicted median time (in minutes) from crest to successful passage [± 95% credible interval (CI)] for each treatment and the control, generated by a Bayesian generalised model.

Treatment combination	Predicted time [95% CI]
Control	5.36 [3.29, 7.46]
Sloped descending	7.75 [5.17, 10.85]
Sloped ascending	4.32 [2.88, 6.08]
Curved descending	2.90 [2.08, 4.40]
Curved ascending	1.74 [1.23, 2.69]

Treatment contrasts	Contrast [95% CI]
Control – sloped descending	−0.37 [−0.91, 0.11]
Control – sloped ascending	0.21 [−0.32, 0.69]
Control – curved descending	0.61 [−0.03, 1.05]
Control – curved ascending	1.12 [0.47, 1.57]

*Note*: Contrasts are reported on the link scale.

## 4 DISCUSSION

The installation of eel passes is a widely employed conservation tool to enable juvenile European eel to ascend riverine barriers that could otherwise delay or halt eel migration. Optimising eel pass design is therefore of high importance in the drive to restore stocks of this critically endangered species and contribute to policy, conservation and management goals. Within an experimental set‐up that simulated ‘up‐and‐over’ type pumped passes (i.e. those passes outside of river flow), tests revealed differences in both the passage efficiency and transit times of juvenile eel ascending various crest shapes and flow conditions. Results indicate that relatively minor modifications to the crest design of pumped passes have the potential to enhance passage efficiency and reduce migratory delay.

Overall, the proportion of eel successfully navigating the pass up and over the crest by the end of the trial was not affected by crest shape or crest flow direction. However, analysis of eel behaviour through video footage revealed variations between passes in the success rate of passage attempts on the crest itself. In three of the treatments (sloped ascending, curved descending and curved ascending) the proportion of successful attempts (i.e., passage efficiency) significantly exceeded 50%, which was not the case for the control and sloped descending treatments. The latter two configurations are the most commonly installed for this pass type (Rosewarne & Wright, 2024), so although treatment effects were small, it is concerning that the two configurations found to be the least efficient in the current study represent the current real‐world status quo. Passage efficiency and success were highly variable throughout the study (Figures 4 and 5), consistent with findings from previous work monitoring juvenile eel passage performance on in situ eel passes. For example, during investigations analysing different ascending slope shapes and substrates on eel passes, Piper et al. (2023) found that juvenile eel percentage success ranged from 0% to 89% across the trials. Vowles et al. (2015) found passage efficiency ranged from 0% to 67% depending on stud presence and configuration on the ascent slope, and Watz et al. (2019) found success rate of juvenile eel varied from 5% to 40% depending on pass substrate.

Attempt failure occurred due to eel approaching the crest and either volitionally turning and descending or being non‐volitionally washed down the ascent ramp. The presence of a crest structure, as opposed to an abrupt end to the ascent ramp (control), reduced the likelihood of this happening. The direction of flow at the crest also appeared to influence eel behaviour, with ascending flow generally outperforming descending flow. The exact mechanism underlying this finding is unclear, although it has been hypothesised that discontinuity of water flow direction at the crest could disrupt rheotactic cues used by migrating eel to navigate upstream, causing eel to turn back from their upstream trajectory (Rosewarne & Wright, 2024). Analysis of transit times in the crest section of passes in the current study provides some evidence for this. The treatment with a curved crest shape and ascending flow performed best, with eel taking ~3 min less to navigate the crest structure compared to the control. In essence, an ascending crest flow provides a continuation of flow conditions experienced by eel during ascent of the ramp, whereas a descending flow presents eel with a contrast (Figure 2). The former may have reduced the potential confusion caused by completing flow cues at the crest, thereby reducing transit time in this zone. Our finding of no significant effect of crest and flow combinations on transit time from the approach line to the crest highlights the role of flow in driving eel behaviour at the crest because flow conditions on this part of the pass should have been consistent across all treatments and the control. Also worthy of consideration is that the two crest shapes tested may have produced different points of no return, that is, the point beyond which an eel could not physically move against the flow and reject.

Migration in diadromous fish is often a time‐limited process (Castro‐Santos et al., 2017; Castro‐Santos & Letcher, 2010). As such, transit time is an important design consideration, with the most effective facilities minimising the time it takes a fish to pass (Haro et al., 2016; Silva et al., 2016). In this study, although transit time differences were small, during migration eel must often navigate multiple barrier structures (Drouineau et al., 2018; Jones et al., 2019). Even a small increase in the energy expenditure required to navigate a pass can, therefore, have significant cumulative effects and potentially compromise energy reserves and onward migration (Du Bureau Colombier et al., 2007; Edeline et al., 2006). Further, optimising transit time reduces additional pressures such as predation (Garcia Leaniz, 2008; Norrgård et al., 2013; Trancart et al., 2020). Migration delays have been shown to have negative impacts, including alterations in reproduction, survivorship and behaviour in some fish species, such as twaite shad (*Alosa fallax*) (Castro‐Santos & Letcher, 2010) and salmonids (Leeuwen et al., 2016; Marschall et al., 2011). However, how delays affect individual fitness, survivorship and population viability is unknown in many species, including European eel (Verhelst et al., 2021). Observations suggested that delays at the crest often arose from increased exploratory behaviour, with eel moving around the approach to the crest area and displaying investigative behaviour over the crest line. An interesting next step would be to conduct more in‐depth behavioural analysis, including studies on energetics, of individuals at the crest, especially if combined with different flow regimes, flow mapping/modelling, to understand what is driving transit time on eel passes and the impact this may have on energy consumption in this species.

It should be noted that eel used for the trials were caught in a trap, having successfully navigated an eel pass similar to the ones tested, which potentially biased our results by selecting for eel with preferential climbing abilities (Podgorniak et al., 2016). This is not of major concern because the study was primarily designed to quantify between treatment differences, although it should be borne in mind when interpreting absolute measures of passage efficiency and transit times. This study was conducted during daytime hours but under dark conditions. Juvenile eel behaviour is strongly influenced by light levels, with an increase in activity during the first hours of darkness, although they do also migrate during the day (Harrison et al., 2014 and references there in). To reduce hold time in the laboratory, test subjects were not acclimated to the adjusted light regime over an extended period. However, the literature on this topic suggests that darkness (either through night‐time or highly turbid water) is the cue eels are predominantly responding too, not night‐time per se. The hold time of test subjects and its potential effects on their performance is an important consideration for laboratory studies and has been shown to alter fish passage previously. Castro‐Santos (2004) found that increased hold time reduced modelled passage times, but decreased maximum swimming distance, in fish passage models of white sucker (*Catostomus commersoni*) and walleye (*Stizostedion vitreum*). Hold time has also impacted experimental passage performance in white sturgeon (*Acipenser transmontanus*) (Cocherell et al., 2011), brook trout (*Salvelinus fontinalis*) and brown trout (*Salmo trutta*) (Duguay et al., 2019), but not in sauger (*Sander canadensis*) (Dockery et al., 2017). The current study limited the hold time to 7 days on‐site, with trials run after a 24‐h acclimatisation period. We found that eel held on site for longer were less likely to navigate the eel pass successfully. One hypothesis is that holding eel in tanks without flow reduces their sensitivity to rheotactic migratory cues, making them less responsive to the flow cues employed to stimulate climbing in the experimental passes. Considering previous findings on hold time, the effects could be species specific and, accordingly, species‐specific holding thresholds should be assessed, or, at a minimum, included as fixed effects, when conducting in situ studies on fish passage performance.

Our results show the importance of validating and quantifying all components of eel passage facilities. Inefficiencies derived from pass sections not being optimised could have wide impacts, particularly when eel must navigate multiple passes during their migration. To our knowledge, no previous study has specifically quantified the efficiency of the crest section of upstream technical eel passes. The installation or modification of the crest section on current eel passages represents a viable low‐cost and theoretically low‐maintenance option to improve migration of the European eel and improve overall river habitat connectivity. Installing curved crest shapes with ascending flow regimes onto current passes would significantly increase the speed at which juvenile eel navigate the structure, with potential reduction in the energy required of migrating juvenile eel to achieve successful passage. However, laboratory set‐ups are smaller and usually more simplistic than conditions in the wild (Salena et al., 2021). As such, field experiments evaluating curved crest shapes and ascending flow on eel passes in situ are desirable as next steps to maximise ecological realism and to gain further evidence on optimising design criteria. Ultimately, this information can be used to improve recruitment for this critically endangered species.

## AUTHOR CONTRIBUTIONS

Michael J. Williamson: investigation (supporting), formal analysis (supporting), data curation (supporting), methodology (supporting), visualisation (supporting), writing – original draft (lead) and writing – review and editing (equal). Bryony E. Allen: conceptualisation (supporting), investigation (equal), data curation (lead), visualisation (supporting), writing – original draft (supporting) and writing – review and editing (supporting). Jack A. Brand: formal analysis (leading), methodology (supporting) and writing – review and editing (supporting). Charlotte Pike: investigation (equal) and writing – review and editing (supporting). Chris Sergeant: investigation (equal) and writing – review and editing (supporting). Chris Grzesiok: methodology (supporting), visualisation (leading) and writing – review and editing (supporting). Rosalind M. Wright: conceptualisation (equal), funding acquisition (equal), methodology (equal), resources (equal) and writing – review and editing (supporting). Adam T. Piper: conceptualisation (lead), investigation (equal), methodology (lead), funding acquisition (equal), resources (equal), supervision (lead) and writing – review and editing (equal).

## FUNDING INFORMATION

This research was principally funded by the Environment Agency. Adam Piper was partly funded by Research England.

## CONFLICT OF INTEREST STATEMENT

The funders had no role in the design of the study; in the collection, analyses or interpretation of data; in the writing of the manuscript; or in the decision to publish the results.

## Supporting information


**Data S1.** Supporting information.

## Data Availability

Raw data supporting the results, and the final code for analysis, are available from the Zenondo Digital Repository: https://zenodo.org/records/13745200.
