# Supplementary material for: Improving eel pass efficiency: The role of crest shape and water flow in facilitating upstream juvenile eel migration
Source: J Fish Biol. 2025 Apr 11;107(2):480–92. doi: 10.1111/jfb.70017 (PMC12360135; doi:10.1111/jfb.70017)
Supplement: Supplementary file 1 — Data S1. Supporting information. [file JFB-107-480-s001.docx]

**Supporting Information**

**Improving eel pass efficiency: the role of crest shape and water flow in facilitating upstream juvenile eel migration.**

Michael J. Williamson^1,2^, Bryony E. Allen^1^, Jack A. Brand^1,3^, Charlotte Pike^4^, Chris Sergeant^1^, Chris Grzesiok^5^, Rosalind M. Wright^6^ & Adam Piper^1,2^

^1^Institute of Zoology, Zoological Society of London, London, UK

^2^Department of Genetics, Evolution and Environment, University College London, London, UK

^3^Department of Wildlife, Fish, and Environmental Studies, Swedish University of Agricultural Sciences, Umeå, Sweden

^4^Conservation and Policy, Zoological Society of London, London, UK

^5^Environment Agency, Sentinel House, Fradley, Staffordshire, UK

^6^Environment Agency, Rivers House, Feering, Essex, UK

**This supplementary material contains:**

**Appendix S1 – Experimental set up**

**Table S1** Predicted median time (in minutes) from approach to crest (± 95% credible interval, CI) for each treatment and the control, generated by a Bayesian generalised model. Note: contrasts are reported on the link scale.

**Figure S1**. Model coefficients from the models of (A) attempt success, (B) passage efficiency, (C) transit time from approach to crest, and (D) transit time from crest to success. Estimates represent posterior medians, with error bars denoting 95% credible intervals. Note: parameter estimates are plotted on the link scale and represent deviations from the intercept.

**Appendix S1 – Experimental setup**

Each pass comprised of: a) an acclimatisation tank (W 32.5 cm; D 35.5 cm; L 21.5 cm, water depth 24 cm), b) a sloped ascent ramp (W 30.2 cm, L 100 cm, 30˚ longitudinal slope), c) a crest, and d) a collection pot (Fig. 1). The entrance to the ascent ramp was submerged 5 cm below the water level and a removable mesh gate prevented eel from leaving the acclimatisation tank prior to trial commencement. The ascent ramp was fitted with a climbing substrate of nylon bristle clusters with 22 mm spacing (Cottam Brush Ltd, UK). Bristles are a common choice of climbing material; a survey of installed eel passes in the UK found 61% had bristle substrate (Rosewarne and Wright, 2024), and small eel (< 200 mm length) favour a dense substrate (20 mm spacing) (Moriarty and Dekker, 1997; Environment Agency, 2011; Rosewarne and Wright, 2024).

Water from the sump beneath each pass was recirculated via an external pump (HZS-200) and delivered to the top of the pass, where it formed: (a) a conveyance flow down the ascent ramp and (b) a crest flow. The conveyance flow was administered by two flexible pipes (1/2" System, Jeton, Taiwan) regulated by a flow splitter arrangement with adjustable valves. This flow was distributed evenly (i.e., wetting the full width of the bristle board ramp) at a rate of 6 L min^-1^, which was deemed sufficient to stimulate climbing while minimising washback of juvenile eel (Solomon and Beach, 2007; Jellyman *et al.*, 2017).

There were three crest flow types: control, descending, and ascending, created by adjusting the direction of three pipes delivering water to the crest at a constant total discharge of 15 L min^-1^. The pipe outlets were furnished with flow-dispersing nozzles, which spread the water evenly across the pass width. The control crest flow was angled directly downwards onto the crest apex. The descending crest flow was directed onto the crest apex and angled slightly to flow down the descent section. The ascending crest flow was directed up towards the apex but did not pass over it onto the ascent ramp, thereby maintaining an overall downstream flow into the collection pot. The crest flow rate was monitored using a digital flow sensor (FD-P20, Keyence), and recorded at the start and end of each trial. Water in the sumps supplying the passes was maintained at 17°C, recorded at the start and end of each trial, and replaced by 10% between trials.

**Figure S1**. Model coefficients from the models of (A) attempt success, (B) passage efficiency, (C) transit time from approach to crest, and (D) transit time from crest to success. Estimates represent posterior medians, with error bars denoting 95% credible intervals. Note: parameter estimates are plotted on the link scale and represent deviations from the intercept.

**
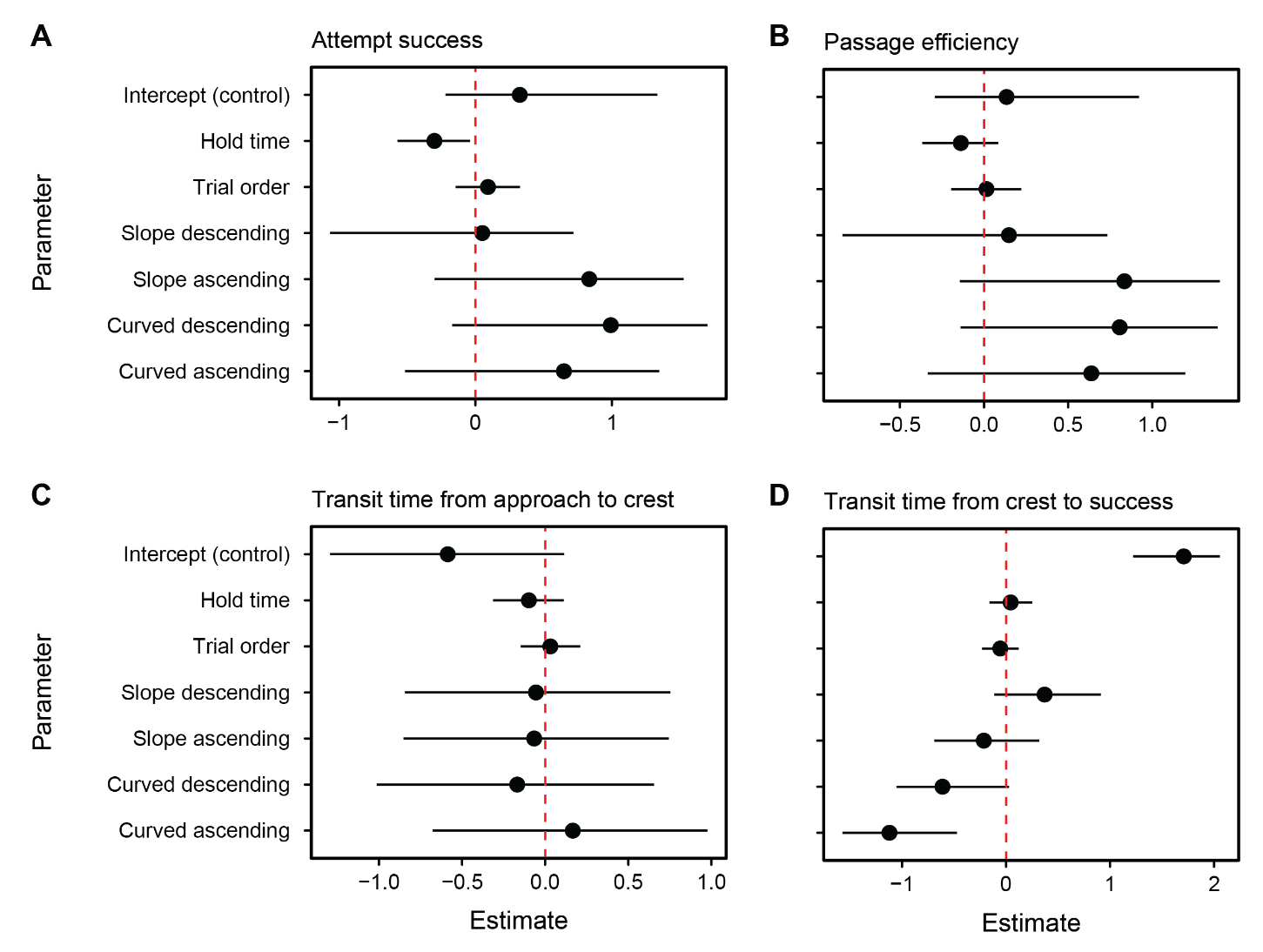
**

**Table S1**. Predicted median time (in minutes) from approach to crest (± 95% credible interval, CI) for each treatment and the control, generated by a Bayesian generalised model. Note: contrasts are reported on the link scale.

| Treatment combination | Predicted time [95% CI] |
| --- | --- |
| Control | 0.57 [0.28, 1.13] |
| Slope descending | 0.54 [0.29, 1.06] |
| Slope ascending | 0.54 [0.28, 1.03] |
| Curved descending | 0.48 [0.25, 0.91] |
| Curved ascending | 0.67 [0.34, 1.26] |
| Treatment contrasts | Contrast [95% CI] |
| Control - Slope descending | 0.06 [-0.75, 0.84] |
| Control - Slope ascending | 0.07 [-0.74, 0.85] |
| Control - Curved descending | 0.17 [-0.65, 1.01] |
| Control - Curved ascending | -0.17 [-0.98, 0.68] |

References

Environment Agency (2011). The Eel Manual. Elver and eel passes: A guide to the design and implementation of passage solutions at weirs, tidal gates and sluices. Bristol.

Jellyman, P., Bauld, J. & Crow, S. (2017). The effect of ramp slope and surface type on the climbing success of shortfin eel (*Anguilla australis*) elvers. *Marine and Freshwater Research* **68**, 1317-1324.

Moriarty, C. & Dekker, W. (1997). Management of the European Eel.

Rosewarne, P. & Wright, R. M. (2024). Eel passage: Improving design and performance SC150001/R1. Bristol, UK: Environment Agency.

Solomon, D. & Beach, M. (2007). Manual for provision of upstream migration facilities for eel and elver. *Science Report SC020075/SR2; Environment Agency: Bristol, UK*.
